# Ring-Expansion/Contraction Radical Crossover Reactions of Cyclic Alkoxyamines: A Mechanism for Ring Expansion-Controlled Radical Polymerization

**DOI:** 10.3390/polym10060638

**Published:** 2018-06-08

**Authors:** Atsushi Narumi, Tetsuya Kobayashi, Masatsugu Yamada, Wolfgang H. Binder, Keigo Matsuda, Montaser Shaykoon Ahmed Shaykoon, Kazushi Enomoto, Moriya Kikuchi, Seigou Kawaguchi

**Affiliations:** 1 Department of Organic Materials Science, Graduate School of Organic Materials Science, Yamagata University, Jonan 4-3-16, Yonezawa 992-8510, Japan; ttc78981@st.yamagata-u.ac.jp (T.K.); yamadasa007@gmail.com (M.Y.); monoceutical@yahoo.com (M.S.A.S.); kazushi.enomoto@riken.jp (K.E.); skawagu@yz.yamagata-u.ac.jp (S.K.); 2 Chair of Macromolecular Chemistry, Faculty of Natural Science II (Chemistry, Physics and Mathematics), Martin-Luther University Halle-Wittenberg, Von-Danckelmann-Platz 4, D-06120 Halle (Saale), Germany; wolfgang.binder@chemie.uni-halle.de; 3 Department of Chemistry and Chemical Engineering, Graduate School of Science and Engineering, Yamagata University, Jonan 4-3-16, Yonezawa 992-8510, Japan; matsuda@yz.yamagata-u.ac.jp; 4 Department of Polymeric and Organic Materials Engineering, Faculty of Engineering, Yamagata University, Jonan 4-3-16, Yonezawa 992-8510, Japan; m-kikuchi@yz.yamagata-u.ac.jp

**Keywords:** cyclic alkoxyamine, radical crossover reaction, ring-expansion reaction, ring-contraction reaction, ring-expansion vinyl polymerization, living radical polymerization, nitroxide-mediated controlled radical polymerization (NMP), macrocyclic polymer, cyclic topology

## Abstract

Macrocyclic polymers present an important class of macromolecules, displaying the reduced radius of gyration or impossibility to entangle. A rare approach for their synthesis is the ring expansion-controlled radical “vinyl” polymerization, starting from a cyclic alkoxyamine. We here describe ring-expansion radical crossover reactions of cyclic alkoxyamines which run in parallel to chain-propagation reactions in the polymerization system. The radical crossover reactions extensively occurred at 105–125 °C, eventually producing high molecular weight polymers with multiple inherent dynamic covalent bonds (NOC bonds). A subsequent ring-contraction radical crossover reaction and the second ring-expansion radical crossover reaction are also described. The major products for the respective three stages were shown to possess cyclic morphologies by the molecular weight profiles and the residual ratios for the NOC bonds (φ in %). In particular, the high φ values ranging from ca. 80% to 98% were achieved for this cyclic alkoxyamine system. This result verifies the high availability of this system as a tool demonstrating the ring-expansion “vinyl” polymerization that allows them to produce macrocyclic polymers via a one-step vinyl polymerization.

## 1. Introduction

**Scheme 1:**
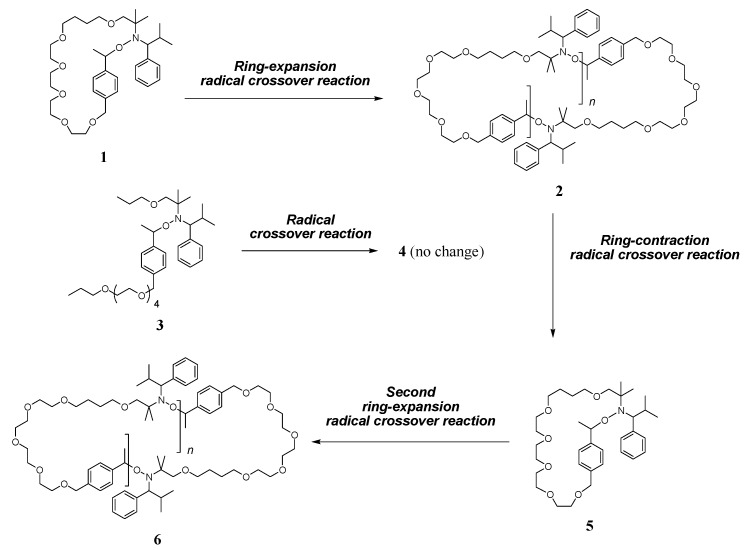
Synthetic pathways of this paper starting from the heating reactions of cyclic and acyclic alkoxyamines together with the structures of the main products for the respective stages.

Considerable attention has been payed to macromolecular architectures containing cyclic topologies [1,2,3,4,5,6,7,8,9,10,11,12,13,14,15,16,17,18,19,20,21,22], where the progress in the synthetic methods for cyclic polymers including ring-closing and/or ring-expansion methods has greatly contributed to the field [23,24,25,26,27,28,29,30,31]. However, ring-expansion “vinyl” polymerization as a tool to generate cyclic polymers via a one-step vinyl polymerization is relatively unexplored, including those based on the nitroxide-mediated controlled radical polymerization (NMP) [32,33,34,35], reversible addition fragmentation chain transfer (RAFT) polymerization [36], living cationic polymerization [37,38,39,40], and others [41]. We have recently developed intramolecularly-tethered alkoxyamine derivatives as cyclic NMP initiators [34,35], envisioning to achieve the synthesis of cyclic, vinyl-based polymers. In particular, styrene was polymerized with a cyclic NMP initiator, and the morphologies of the resulting high molecular weight polymers were discussed in terms of their radius of gyrations [35]. The reaction system is, however, not fully characterized primarily due to the occurrence of radical ring-crossover reactions, running in parallel to the conventional chain-growth propagation reactions, which in consequence lead to non-uniform polymer chain constructions. Thus, it is of great interest to exclude the vinyl polymerization reaction from the system and verify the “ring-expansion radical crossover reaction”. We here report on methodology to understand the ring-crossover reactions taking place during the polymerization systems with cyclic NMP initiators. We expect that simplified information on this process is provided from the structural analyses of the products obtained by radical crossover reactions because the molecular weight profiles and spectroscopic data become clearer due to the absence of the large polymer units constructed by vinyl polymerizations. As displayed in Scheme 1, cyclic alkoxyamine **1** is heated in the absence of any polymerizable vinyl monomer at the appropriate temperatures where NMP-polymerization is normally taking place. The reactions of **1** are performed using diverse conditions and the structures of the resulting polymer **2** are characterized to clarify the temperature effects and time-dependent changes. In this paper, we further report the heating reaction of polymer **2** under highly diluted conditions using a solvent to demonstrate the “ring-contract radical crossover reaction”. Finally, the capability for “the second ring-expansion radical crossover reaction” is explored to ensure the presence of cyclic morphologies in polymer **2**.

## 2. Materials and Methods

### 2.1. Materials

Cyclic alkoxyamine **1** was prepared according to the literature [35]. Other materials were obtained from commercial sources and used as received unless otherwise stated.

### 2.2. Methods

The ^1^H and ^13^C NMR spectra were recorded using a JEOL JNM-ECX400 instrument (Tokyo, Japan). The size exclusion chromatography (SEC) analysis was performed using a system equipped with a Shodex GPC-System 21 (Tokyo, Japan), a SYSTEM INSTRUMENTS WP-03 Plus pump (Hachioji, Japan), a Shodex DEGAS degasser, Shodex KF-806L (8.0 mm × 300 mm, average bead size: 10 μm, exclusion limit: 2 × 10^4^ kg·mol^−1^), KF-804L (8.0 mm × 300 mm, average bead size: 7 μm, exclusion limit: 4 × 10^3^ kg·mol^−1^), K-803 (8.0 mm × 300 mm, average bead size: 6 μm, exclusion limit: 70 kg·mol^−1^) columns, a Shodex RI-71S, and a TOSOH UV-802 using (Tokyo, Japan) THF as the eluent at a flow rate of 1.0 mL·min^−1^ at 40 °C. The weight average molecular weight (*M*_w_), the molecular weight distribution (*M*_w_/*M*_n_), and the molecular weight at the peak top (*M*_p_) were determined by the RI based on linear PSt standards (Tosoh Co., Tokyo, Japan) with the *M*_w_s of 775,000, 422,000, 186,000, 114,000, 44,100, 16,700, 8300, 5120, 2360, 870, 500, and 110.

### 2.3. Ring-Expansion Radical Crossover Reaction (Synthesis of **2**)

Cyclic alkoxyamine **1** (21 mg, 35 μmol) was placed in a 2 mL dry glass ampule and then degassed *in vacuo*. After being flame-sealed under a vacuum, the ampule was allowed to stand at 115 °C for 3 h. The reaction was stopped by rapid cooling with liquid nitrogen to produce polymer **2-V** as a pale yellow viscous liquid (recovered yield, 20 mg; 93%). Data for **2-V**: ^1^H NMR (500 MHz, CDCl_3_): δ 7.45–7.14 (m, phenyl-*H*), 4.90 (m, C*H*ON), 4.57 (OC*H*_2_-phenyl), 3.64–2.31 (br, OC*H*_2_C*H*_2_O, OC*H*_2_(CH_2_)_2_C*H*_2_O, NC*H*, OCH_2_(C*H*_2_)_2_CH_2_O), OC*H*_2_C(CH_3_)_2_N and C*H*(CH_3_)_2_), 1.67–0.20 (br, C*H*_3_CH(-phenyl)O, CH(C*H*_3_)_2_ and NC(C*H*_3_)_2_). *M*_w_ = 38,000, *M*_w_/*M*_n_ = 24.8. *M*_p_ = 58,400.

### 2.4. Ring-Contraction Radical Crossover Reaction (Synthesis of **5**)

A mixture of **2-V** (19 mg) and 1,3-dichlorobenzene (8.6 mL) was placed in a dry glass ampule with a magnetic stir bar and then degassed by five freeze−evacuate−thaw cycles. After being flame-sealed under a vacuum, the ampule was stirred at 115 °C for 9 h. The polymerization was stopped by rapid cooling with liquid nitrogen. The mixture was evaporated to dryness to give product **5-V** as a yellow oil (recovered yield, 14 mg; 77%). Data for **5-V**: ^1^H NMR (500 MHz, CDCl_3_): δ 7.44–7.15 (m, phenyl-*H*), 4.91 (m, C*H*ON), 4.57 (OC*H*_2_-phenyl), 3.67–2.33 (m, OC*H*_2_C*H*_2_O, OC*H*_2_(CH_2_)_2_C*H*_2_O, NC*H*, OCH_2_(C*H*_2_)_2_CH_2_O, OC*H*_2_C(CH_3_)_2_N and C*H*(CH_3_)_2_), 1.63–0.18 (m, C*H*_3_CH(-phenyl)O, CH(C*H*_3_)_2_ and NC(C*H*_3_)_2_). *M*_w_ = 690, *M*_w_/*M*_n_ = 1.87. *M*_p_ = 231.

### 2.5. Second Ring-Expansion Radical Crossover Reaction (Synthesis of **6**)

The same procedure as that for the synthesis of **2** was applied for **5-V** (14 mg) to give **6-V** (recovered yield, 11 mg; 78%) Data for **6-V**: ^1^H NMR (500 MHz, CDCl_3_): δ 7.45–7.15 (m, phenyl-*H*), 4.90 (m, C*H*ON), 4.55 (OC*H*_2_-phenyl), 3.65–2.32 (m, OC*H*_2_C*H*_2_O, OC*H*_2_(CH_2_)_2_C*H*_2_O, NC*H*, OCH_2_(C*H*_2_)_2_CH_2_O, OC*H*_2_C(CH_3_)_2_N, C*H*(CH_3_)_2_), 1.61–0.20 (m, C*H*_3_CH(-phenyl)O, CH(C*H*_3_)_2_ and NC(C*H*_3_)_2_). *M*_w_ = 13,500, *M*_w_/*M*_n_ = 6.59. *M*_p_ = 14,700.

## 3. Results and Discussion

### 3.1. Ring-Expansion Radical Crossover Reaction

#### 3.1.1. Temperature Effect

**Figure 1:**
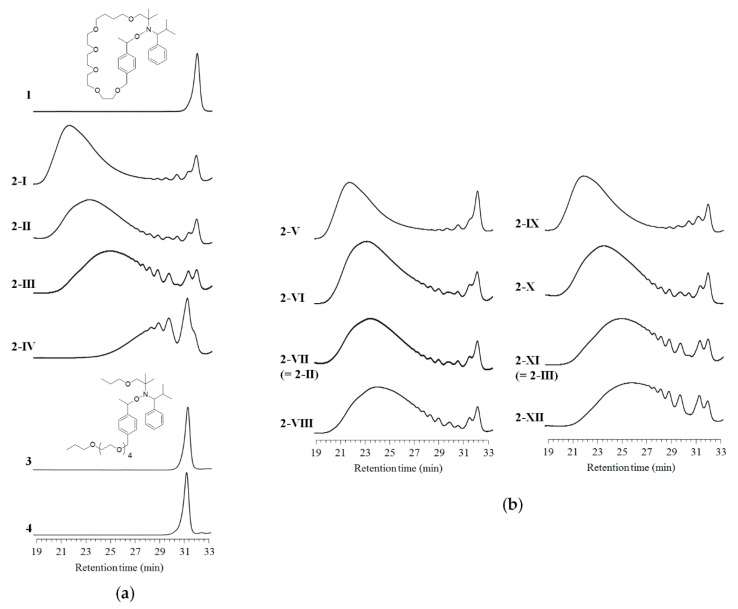
SEC traces of (**a**) **1**, **2-I~IV**, **3**, and **4** and (**b**) **2-V**~**VIII** and **2-IX**~**XII**.

Cyclic alkoxyamine **1** was heated in the bulk condition without any vinyl monomers (Scheme 1). The reactions were performed at diverse temperatures such as 105, 115, 125, and 135 °C for 12 h to afford products **2-I**, **2-II**, **2-III**, and **2-IV**, respectively (entries 1–4 in Table 1). Figure 1a shows size exclusion chromatography (SEC) traces of **1** and products **2-I**~**IV**. The trace of **1** exhibited a sharp peak at the retention time (RT) of 32 min. The traces of **2-I**~**III** are first described, which showed the broad peaks due to high molecular weight polymer species at the RTs ranging from 19 to 27 min together with the multiple peaks due to oligomeric species at the RTs ranging from 27 to 30 min. The peaks attributable to monomeric species were observed at the RTs of 31 and 32 min as will be described later in the paper. Thus, the extensive occurrences of radical ring-crossover reactions were clearly revealed, which would be featured phenomena for alkoxyamine derivatives with cyclic morphologies.

The SEC measurement provided the weight average molecular weight (*M*_w_) and the molecular weight distribution (*M*_w_/*M*_n_) based on a calibration using linear PSt standards. As listed in Table 1, the *M*_w_ values ranged from 13,600 to 42,800 and *M*_w_/*M*_n_ values ranged from 7.04 to 18.1 for **2-I**~**III** (entries 1–3). The molecular weight at the peak top (*M*_p_) would be the appropriate value to discuss the tendencies for this system showing multimodal SEC profiles. The *M*_p_ values for the main products were 57,100 for **2-I**, 24,900 for **2-II**, and 12,400 for **2-III**. As the molecular weight of **1** is 602, the respective *M*_p_ values corresponded to the 65-, 30-, and 15-mers. However, this estimation is very rough because the *M*_p_ values were linear PSt-based apparent ones, whereas again supports the extensive occurrences of radical ring-crossover reactions. The *M*_p_ values tended to decrease as the reaction temperatures increased, as shown in Figure 1a. The **2-IV** system (Figure 1a and entry 4 in Table 1) showed a different feature as compared to the others in which the major peak was observed in the low molecular weight regions at the RTs ranging from 30 to 32 min. We previously reported that acyclic alkoxyamine **3** (Scheme 1) was prepared as a linear counterpart of **1** and used as an initiator for the polymerization of styrene [36]. In this study, we newly performed the heating reaction of **3** to give product **4** (Entry 5 in Table 1). Figure 1a shows the SEC traces of **3** and **4** in which both exhibited sharp peaks at the RT of 31 min. Hence, no significant change was observed for the molecular weight profiles for the **3** system. It should be stated that the result that the RT of **3** (31 min) is shorter than that of **1** (32 min) is rationalized by a compact cyclic structure of **1**. Furthermore, this RT difference is advantageous for the mechanism clarification. For the **2-I**~**IV** systems, the peaks were observed at the RTs of 31 and 32 min (Figure 1a) and we assigned the respective peaks to those for the linear (cleaved) unimer and the cyclic (tethered) one.

**Figure 2:**
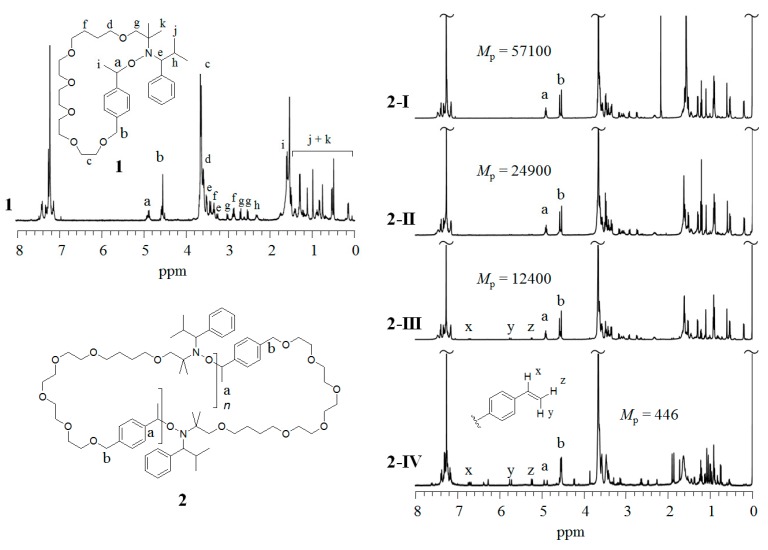
^1^H NMR spectra of **1** and **2-I**~**IV**.

Figure 2 shows the ^1^H NMR spectra of **1** and **2-I**~**IV**. The characteristic signal due to the methine proton in the alkoxyamine moieties (a, 1H) appeared at 4.9 ppm for the spectrum of **1**. The fact that the corresponding signals were observed for **2-I**~**2-III** suggested the presence of dynamic covalent bonds (NOC bonds) even after the reactions. The signal due to the methylene protons in the benzyl position (b, 2H) was also observed at 4.5 ppm for the spectrum of **1**. We determined the *I*_a_/*I*_b_ values where the *I*_a_ and *I*_b_ denote the integrations (peak areas) of the signals (a) and (b), respectively. The *I*_a_/*I*_b_ values were 1.0/2.0 for **1**, 0.93/2.0 for **2-I**, 0.90/2.0 for **2-II**, and 0.74/2.0 for **2-III**. The *I*_a_/*I*_b_ values would be equivalent to the residual ratios for NOC bonds (φ in %). The φ values were 93% for **2-I**, 90% for **2-II**, and 74% for **2-III** as listed in entries 1-3 in Table 1. Thus, a large amount of NOC bonds remained in the products obtained at the reaction temperatures of 105–125 °C. On the other hand, for **2-IV** obtained at the temperature of 135 °C, the occurrences of side reactions were suggested by the NMR analysis. The signals (x) at 6.7 ppm, (y) at 5.7 ppm, and (z) at 5.2 ppm appeared for the spectrum of **2-IV** (Figure 2). As judged from the chemical shifts and coupling constants, the signals were assignable to the vinyl protons formed as a result of irreversible heterolysis (disproportionation) of the NOC bonds. The similar signals were also observed for **2-III** (Figure 2), whereas their intensities were very weak. We reached the conclusion that “ring-expansion radical crossover reaction” extensively occurred at 105–125 °C, eventually producing high molecular weight polymers with inherent multiple NOC bonds as shown in Scheme 1.

#### 3.1.2. Time-Dependent Changes

The reactions of **1** were performed for different times such as 3, 6, 12, and 24 h to elucidate time-dependent changes. Figure 1b shows the SEC traces of the products **2-V**~**VIII** and **2-IX**~**XII**, which were obtained for the 115 and 125 °C systems, respectively. Table 1 summarizes the results for the characterizations of **2-V**~**VIII** (Entries 6–9) and **2-IX**~**XII** (Entries 10–13). The *M*_w_ values ranged from 10,300 to 38,600. The *M*_w_/*M*_n_ values were between 6.11 and 24.8. The *M*_p_ values ranged from 9940 to 58,400. Thus, high molecular weight polymers were formed as a result of the radical ring-crossover reactions. The *φ* values varied from 64% to 98%. In general, both the *M*_p_ and φ values were high for the systems at 115 °C as compared to those at 125 °C (Table 1); for example, the highest values were *M*_p_ = 58,400 and φ = 98% for **2-V**. It should be noticed that the SEC trace showed the peaks at the RT of 32 min (Figure 1b). Thus, **2-V**~**XII** include considerable amounts of cyclic (tethered) unimers. The traces also exhibited the peak or shoulder attributable to linear (cleaved) unimers at the RT of 31 min. As judged from the peak intensities, the cyclic unimers were preferentially formed over the linear ones throughout the reactions for the 115 °C system, while the formation of the linear ones increased with the increasing reaction times for the 125 °C system. This result supported that 115 °C is a suitable temperature in the view point of preventing ring cleavages.

### 3.2. Ring-Contraction Radical Crossover Reaction

**Figure 3:**
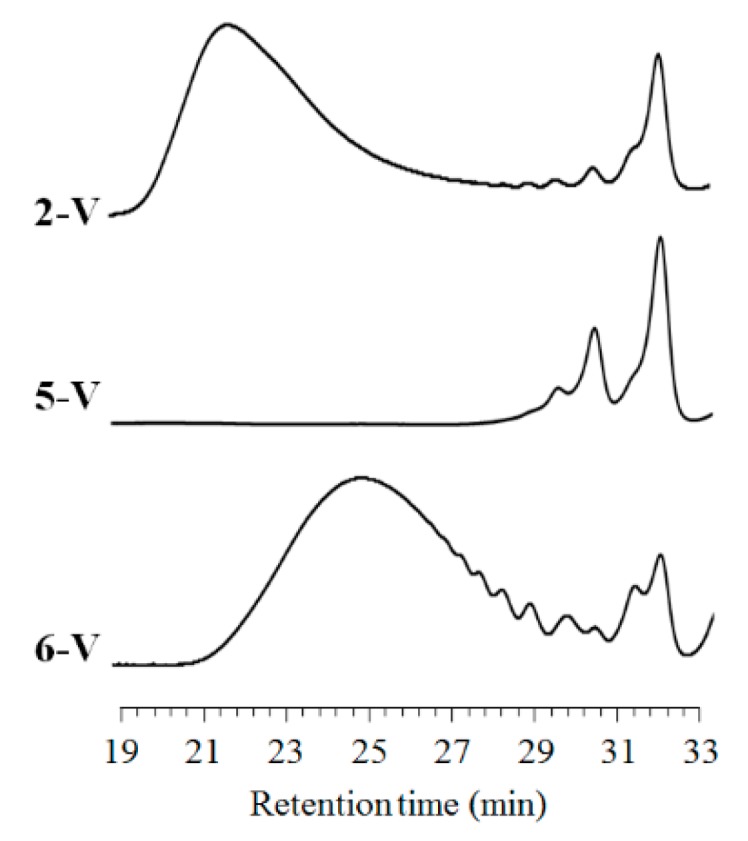
SEC traces of **2-V**, **5-V**, and **6-V**.

In order to ensure the performance of the cyclic alkoxyamine system, we exploited the “ring-contraction radical crossover reaction” (Scheme 1). Polymer **2-V** with the *M*_p_ for the main peak of 58,400 was heated at 115 °C in 1,3-dichlorobenzene under a highly diluted condition such as 0.003 M (Entry 14 in Table 2). The reaction for 9 h produced a product **5-V**. Figure 3 shows the SEC trace of **5-V** with the *M*_w_(*M*_w_/*M*_n_) of 690(1.87) and the *M*_p_ for the main peak of 231. The trace exhibited multimodal peaks only in the low molecular weight regions with the RTs ranging from 29 to 32 min and the main peak with the RT of 32 min was assigned to a cyclic (tethered) unimer. The NOC bonds remained with the high φ value of 90%. Similar results for these ring-expansion/contraction phenomena were previously reported by Otsuka and coworkers [42]. They described the results using phrases “polymerization of macrocyclic alkoxyamine” and “depolymerization of poly(alkoxyamine)” in the paper, where they have studied the dynamic covalent polymer systems based on the 2,2,6,6-tetramethylpiperidine-1-oxyl (TEMPO) as persistent radicals.

**Scheme 2:**
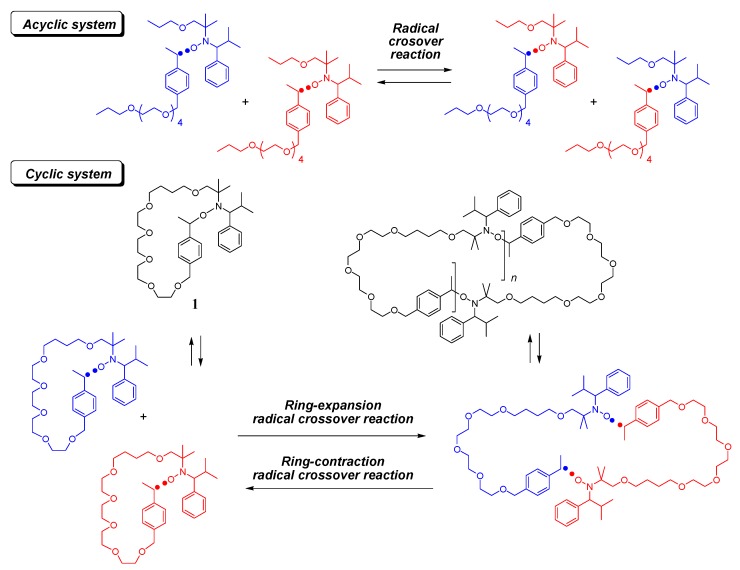
Radical crossover reactions for the acyclic and cyclic systems.

We here summarize radical crossover reactions for the cyclic systems, which significantly differs from those for the acyclic system. Scheme 2 shows the radical reactions starting from two kinds of alkoxyamines in this study. No change has been brought by the intermolecular radical crossover reactions for the acyclic system in terms of molecular weights and morphologies as experimentally shown in this study. The applications based on the radical exchange reactions have been discussed elsewhere [43,44,45,46]. On the other hand, for the cyclic system, the inherent dynamic covalent bond (NOC bond) takes place on a homolytic cleavage to generate a reactive benzyl radical and a persistent nitroxide radical (Scheme 2). A subsequent recombination reaction provides opportunities for the NOC bond exchange. The ring-expansion reactions proceed when the intermolecular recombination reactions occur, in which a prime driving force would be a decrease in the ring strain, resulting in the large increase in the molecular weights. This phenomenon is attributable to an inherent NOC bond in a cycle or multiple NOC bonds in a linear chain. The result of this study is originally due to the inherent NOC bond in a cycle because **1** possesses only one dynamic covalent bond. Under highly diluted conditions where the intermolecular reaction would not be accepted, intramolecular recombination would proceed, eventually returning to the original cyclic unimer.

### 3.3. Second Ring-Expansion Radical Crossover Reaction

When taking into account the above-mentioned mechanisms, the result that the main components in **5-V** was not a cleaved unimer but a tethered one with the high φ value of 90% motivated us to demonstrate the “second ring-expansion radical crossover reaction”. We performed the reaction of **5-V** at 115 °C for 3 h in the bulk condition to produce products **6-V** (Entry 15 in Table 2). Figure 3 shows the SEC trace of **6-V** with the *M*_w_(*M*_w_/*M*_n_) of 13,500(6.59) and the *M*_p_ for the main peak of 14,700. The trace again showed the broad peaks due to high molecular weight polymer species at the RTs ranging from 21 to 27 min together with the multiple peaks due to the oligomeric species at the RTs ranging from 27 to 30 min. The monomeric species were also observed at the RTs of 31 and 32 min. Therefore, the featured reactions due to the “cyclic” alkoxyamine system again extensively occurred to produce **6-V** with a high φ value of 84%. This result strongly supports that **2-V** (and also **5-V**) contains polymeric species with cyclic morphologies.

## 4. Conclusions

A series of radical ring-crossover reactions were verified using the strategy that cyclic alkoxyamine and their reaction products were heated at the NMP temperatures without any vinyl monomers. The extensive occurrences of “ring-expansion radical crossover reaction” were clearly revealed, which would be featured phenomena for alkoxyamine derivatives with cyclic morphologies, eventually producing high molecular weight polymers with inherent dynamic covalent bonds. The “ring-contract radical crossover reaction” was performed for the resulting polymer and subsequently the “second ring-expansion radical crossover reaction” was demonstrated, which strongly supported the presence of high amounts of cyclic morphologies throughout the series of reactions.

## Figures and Tables

**Table 1:** Conditions and results for the radical crossover reactions.

Entry	Starting Material	Temp. (°C)	Time (h)	Product Code	*M*_w_^1^	*M*_w_/*M*_n_^1^	*M*_p_^1^	φ ^2^ (%)
1	**1**	105	12	**2-I**	42,800	18.1	57,100	93
2	**1**	115	12	**2-II**	29,600	11.0	24,900	90
3	**1**	125	12	**2-III**	13,600	7.04	12,400	74
4	**1**	135	12	**2-IV**	2930	3.33	446	-
5	**3** (control)	115	12	**4**	506	1.06	426	90
6	**1**	115	3	**2-V**	38,000	24.8	58,400	98
7	**1**	115	6	**2-VI**	26,600	11.3	28,600	92
8 (=2)	**1**	115	12	**2-VII (=2-II)**	29,600	11.0	24,900	90
9	**1**	115	24	**2-VIII**	20,300	8.5	19,300	82
10	**1**	125	3	**2-IX**	38,600	15.9	50,600	92
11	**1**	125	6	**2-X**	23,500	9.27	24,500	84
12 (=3)	**1**	125	12	**2-XI (=2-III)**	13,600	7.04	12,400	74
13	**1**	125	24	**2-XII**	10,300	6.11	9940	64

^1^ Determined by SEC in THF based on the calibrations using linear PSt standards. ^2^ Residual ratios for NOC bonds determined by ^1^H NMR spectra.

**Table 2:** Condition and results for the ring-contraction radical crossover reaction and subsequent second ring-expansion radical crossover reaction.

Entry	Starting Material	Temp. (°C)	Time (h)	Product Code	*M*_w_^1^	*M*_w_/*M*_n_^1^	*M*_p_^1^	φ ^2^ (%)
14	**2-V**	115	9	**5-V**	690	1.87	231	90
15	**5-V**	115	3	**6-V**	13,500	6.59	14,700	84

^1^ Determined by SEC in THF based on the calibrations using linear PSt standards. ^2^ Residual ratios for NOC bonds determined by ^1^H NMR spectra.
